# Supplementary material for: National consensus on entrustable professional activities for competency-based training in anaesthesiology
Source: PLoS One. 2023 Jul 11;18(7):e0288197. doi: 10.1371/journal.pone.0288197 (PMC10335696; doi:10.1371/journal.pone.0288197)
Supplement: S1 Appendix — (PDF) [file pone.0288197.s001.pdf]

S 1 File. Alignment process of the EPAs

The comments provided in the open text fields by the chair directors for each EPA are displayed.

After the content analysis and mapping, n = 42 comments were identified to be relevant for further adaptations. These comments are highlighted in red. A summary of the alignment process and comments of the expert group regarding the 42 comments are provided in the black framed boxes. Note: *The comments are one-to-one translations, disregarding the completeness of sentences.*

EPA: *Administer general anaesthesia including regular airway management*

- Only in the second half of the first year and supervision (attending) needs to be in the vicinity (OR)
- Including the recognizing if the probability of a “regular airway” is high and can be expected
- Maybe a little “small” for an EPA. Airway management would be an DOP for me but with induction of narcosis it should be sufficient
- The EPA represents an elementary basic task and should therefore be entrusted in the first part of the training
- Basic equipment for any anaesthesiologist
- It’s not about training, it’s about further education

- After 6-8 months

EPA: *Providing perioperative care for patients with ASA ≤ III*

- Only in the second half of the first year and supervision (attending) needs to be in the vicinity (OR)
- Requirement: Competency, recognize something out of the ordinary to request backup in time
- **The first EPA airway management could be part of this EPA, then we could save one**
- Also basics, should be in the first year of training
- I would see ASA 2 in the first year, ASA 3 should be in the second year
- After familiarization in the corresponding OR

We would not merge the two EPAs "*Administer general anaesthesia including regular airway management*" and "*Providing perioperative care for patients with ASA ≤ III*" since their educational focus lies on the induction of narcosis and airway management on the one hand and providing perioperative care on the other hand. For one EPA the airway management is the central competency (including lung physiology; ventilation etc). For the other EPA, induction of anaesthesia and providing perioperative care is centered (a.e pharmacology). Therefore, necessary underlying competencies have been defined and integrated into the complete description of each EPA.

EPA: *Providing anaesthetic care for small laparoscopic surgery*

- **Don't break it down in to too small parts with certain procedures**

- Such narcosis are being carried out during the first or second year of training
- Depending on the rotation plan
- Year of training should be depending on the ASA status

The different extended laparoscopies should be considered as different types of surgical procedures, with different underlying hazards. Furthermore, the perioperative care (large vs. small laparoscopic surgery) requires different levels of theoretical knowledge and practical competencies. Therefore, we would not merge the EPAs.

EPA: *Performing a premedication round (preoperative evaluation) including patient education*

- **First or second year: A difficult topic – here should be a smooth transition (longer leach) and encouragement to stay in close contact to an attending in case of doubt reasonable**
- **I think this is very important, because anesthesiologic concepts are being developed here. Goes in the direction of self-responsibility**
- **There needs to be more experience to complete this EPA, so more towards the last years of training**
- ASA 3 patients need to be discussed with a consultant
- ASA 1 and ASA 2 patients. After second year all others and option of supervision at any time

We find this to be an interesting topic since there is a need for an early involvement in patient communication and premedication, but there must also be supervision available since

decisions have a great impact on the treatment plan and care. Based on the comments, that supervision should always be available and the significance of decisions, we decided that this EPA should be merged with the EPA "*Communication with relatives of critically ill patients and consultation about treatment plans*". Both EPAs integrate plenty of overlapping competencies, which can be more effectively covered in one EPA. Another benefit of the merger is the increase of the year of entrustment which gives the learners an adequate period to master the competencies and become competent and mature for (summative) entrustment.

EPA: *Indication and administration of blood transfusion*

- Basics, in the first year of training
- It's not about training, it's about further education
- Performing in the first year, indication in the 3rd year

EPA: *Providing postoperative care in the recovery room*

- Can be done in the middle part
- Consultant within shouting range

EPA: *Indication and performance of analgosedation*

- Analgosedation in the central-OR can be done earlier, in outside-areas=attending qualification
- Also middle part
- The feeling for anaesthetics needs to be there -> sufficient intubation experience
- **Should not be done without defining the ASA status**

We do acknowledge the comment regarding the ASA status. To prevent insufficient preparation and training we implemented additional underlying competencies that require a minimum of adequately performed analgosedations, before entrustment happens. With this multi-step approach towards entrustment (summative), we think that the growing complexity of ill patients and increasing ASA status can be appropriately addressed. Therefore, we would not divide this EPA in different EPAs including the ASA status. The defined competencies and assessments encompass the different complexity levels of analgosedation, integrating the ASA status.

EPA: *Providing postoperative pain management*

- More in the last period of training, should be done after a pain rotation

EPA: *Indication, consideration and performance of spinal and epidural anaesthesia*

- Indication early because of premedication, start with spinal if possible

- **Depending on the ASA status and chosen technique (spinal, peridural, thoracic peridural)**

We think that preexisting conditions (different ASA stauts) are highly relevant for the selection of the technique. We have already included these considerations in the underlying competencies of the EPA, addressing the individual characteristics and conditions of the patients and selecting the proper technique, while acknowledging indications and contraindication. Entrustment should happen in a more complex manner, including all the mentioned considerations, theoretical knowledge and finally the practical skills. Therefore, we would not divide this EPA in different EPAs according to the ASA status.

EPA: *Communication with relatives of critically ill patients and consultation about treatment plans*

- Competencies reached during ICU rotation
- **In principle a useful EPA although you shouldn't overload such a system**
- **Last part of training, here you need experience**
- **Depending on complexity – Limitation of therapy only through an attending**
- Depending on rotation-plan – intensive care unit

We do agree that this is a challenging EPA with lots of underlying communication competencies as well as experience. As discussed in the comment to the EPA "*Performing a premedication round (preoperative evaluation) including patient education*" we merged this EPA due to significant overlapping in competencies. Hereby, we exclude "critical talks" as they

would take place for critical ill patients´ relatives and focus more on communication skills whiler performing a premedication and a postoperative informative communication.

EPA: *Providing anaesthetic care for large laparoscopic surgery*

- Relevance depending on the hospital
- Could be useful, only with too many EPA there must be an individual assessment, therefore …
- Depending on the rotation plan and training facility

EPA: *Providing anaesthetic care for intracranial surgery without increased intracranial pressure*

- This is depending on the training facility. I think that in the midterm we will have more rotations between hospitals. Depending on when which rotation could take place, this EPA could be positioned in the second year
- Is used at our clinic at an early stage

EPA: *Performing in-house transfers of critically ill patients*

- After the ICU-rotation
- High priority at a university hospital
- **DIVI-transfer certification**
- **After rescue service certification and intensive care experience**

We agree that in-house transfers of critically ill patients are high risk tasks which have the risk of adverse events when not conducted properly. The suggested certifications (DIVI-transfer course or rescue certification) offer an additional level of training and knowledge but are mostly suited for inter-hospital transfers. Transferring a patient to the ICU after a surgical procedure is a task that is performed daily by trainees and should therefore be entrusted when a sufficient level of competency is reached- not including a transfer course certification. Nevertheless, we want to emphasize the great value of such trainings.

EPA: *Providing perioperative care for patients with ASA > III*

- More in the last part of training, when integrated in the service structures

EPA: *Indication and performance of a regional anaesthesia technique*

- This is essentially depending on the training of the assistants. If regular training and education is provided this can be done in the second year.
- **The peripheral anaesthesia techniques can be – depending on the difficulty and potential of complications – divided. Example: “easy” standard procedures (Ax. Plex) could be entrusted in the first year, blockages close to the pleura require more skills. Here it will be depending on the caseload of the rotation, in which year it can be entrusted**
- Indication in the first year, performing without supervision in the fourth
- Can be started early in the trauma rotation

- Should be provided as SOP
- **Here should be begun with the axPlex, followed by the ischicadicus in the second and increasing as well as further differentiating in the following years. (prox. Ischiadicus, supraclav. Plex). Depending on the educational system as well as rotations**
- **In which year the regeional anaesthesia should be performed without supervision is depending on the time, at which the technique was learned. Because of that this question can not be really answered. I chose the third year. For some techniques the first year is right, for some the fith.**

We agree with the comments that the different types of techniques require different levels of experience and acquired competencies. This EPA includes the different procedures and focuses (as demanded in the comments) on the relevant competencies- the different procedures are OPAs of this EPA. The OPAs will be entrusted as the learner's progress in their training. After the OPAs are entrusted- then the summative entrustment decision will take place for the EPA. Therefore, we see no need for additional adaptation of this EPA.

EPA: *Administer general anaesthesia in patients with increased risk of aspiration*

- Classic on call anaesthesia
- High risk narcosis, third/fourth year
- For an RSI there is always an attending/consultant (plus resident) useful (for patients safty)

EPA: *Providing anaesthetic management for pregnant patients*

- High risk anaesthesia, last part of training

EPA: *Providing anaesthetic care for thoracic surgery (including lung seperation) with normal lung function*

- Also last part of training
- **Please define normal lung function**
- Depending on the rotation plan

The distinction of “nomal” and “limited” lung function is often based on individual factors depending on the patient, hospital standards or estimation of the physician. Therefore, a clear cutoff between “normal” or “limited” cannot be given but should be subject of further discussions, to evaluate if a national consensus can be reached in terms of the correct definition.

EPA: *Haemodynamic management of major blood loss*

- **At the maximum care hospitals of highest priority -> Simulation ?**
- I wouldn’t deal with this isolated from other high risk areas
- Haemodynamice relevant bleeding in the fourth year

We think that this EPA is of great importance and should not be solely limited to maximum care hospitals. Although major bleeding might occur less frequently in smaller hospitals, the management of major blood loss includes core competencies of an anaesthetist. of the Underlying competencies can be conveyed and assessed with simulation. Therefore, we have integrated simulation scenarios for this EPA. These simulation trainings could take place at maximum care hospitals with the participation of non-maximum care hospitals.

EPA: *Providing anaesthetic care for extensive open abdominal surgery*

- Challenging task in the middle of training
- Depending on rotation plan

EPA: *Administer general anaesthesia including airway management in patients with anticipated difficult airway*

- **Simulation ?**
- Essential, has to be mastered before board certification
- At least ready for board certification standard
- As EPA level 3 better with consultant/attending and direct supervision = level 2
- Depending on rotation plan

We do agree that simulation is a strong and useful instructive design to train the competencies of this EPA. Having simulations and practicing the use of different airway devices as well as

fiberoptic handling can be supportive in reaching educational goals. We offer additional courses at our department to support the trainees on a voluntary basis and would like to encourage other hospitals to do likewise. Considering the limited resources of smaller hospitals, we don't think that simulation should be compulsory but in a next step a program could be offered by maximum care hospitals for smaller hospitals. Such programs would also facilitate networking.

EPA: *Indication of anaesthetic technique and performance of medullary or general anaesthesia for regular and emergency caesarean section*

- **The more urgent the more simulation**
- Before board certification
- **Emergency caesarean section -> board certified, planned in spinal -> in the third year**

We think that the importance of this EPA is appropriately reflected in the results of the validation. The year in which the EPA should be entrusted is 4,0 and the CVI is 1,0. As stated before we do recognize the relevance of simulation for educational purposes in critical situations and think that it can be beneficial to prepare trainees for the competencies of this EPA as well.

EPA: *Administer general anaesthesia in pediatric patients over the age of five*

- Responsible task in the last part of training

EPA: *Indication and performance of ultrasound use and diagnostic (a.e FAST) and therapeutic consequences*

- **Ultrasound should definitely be part of the training! If FAST has the highest relevance? The question is which technique/examination peri- or intraoperative. I would focus on the intraoperative TEE.**
- More in the attending area
- **Better are in depth ultrasound courses**
- Is done by radiology in most cases
- **Ultrasound is important in training, but why (only) FAST**

We think that the discussion around ultrasound in anaesthesia training is very interesting. Due to the results of the validation, we excluded this EPA from the final curriculum because of low approval ratings as well as a low CVI. Interestingly some comments highlight the importance of ultrasound in clinical training, which leads to the assumption that significance of this EPA is perceived differently. We would encourage a further discussion between stakeholders to reach an agreement on the use of ultrasound in training as well as the appropriate training concept (use of national ultrasound courses, EPA, clinical rotations, etc.).

EPA: *Providing anaesthetic care for intracranial surgery with (the risk of) increased intracranial pressure*

- Simulation
- Wouldn't disconnect this from neurosurgical procedures

EPA: *Management of the unanticipated difficult airway*

- **Acquiring the skills should start early (simulator, followed by clinic)**
- **Simulation**
- Unexpected comes often, algorithm needs to be well known, getting help is essential
- Difficult airway as general EPA
- **An unexpected EPA cannot be assigned to a year of training**
- **An unexpected difficult airway cannot be entrusted to anyone. If so then it would be expected.**
- If unexpected difficult airway -> get consultant
- Yearly training

The management of the unanticipated difficult airway is probably one of the most critical and challenging situations of anaesthesia. As provided in the comments and consistent with our opinion, regular training, knowledge of corresponding algorithms and simulation provide a foundation for this rare, but life-threatening event. We don't agree with the statements, that an unexpected difficult airway cannot be entrusted or mapped to a year of training. Encompassing the necessary skills, tools and competencies, combined with regular training, the trainee will become competent in managing this essential EPA. We do not rule out (on the contrary- we endorse!) that help of more experienced colleagues should at hand as soon as possible. Nevertheless, any anaesthetist should have basic knowledge and procedural skills as described in this EPA.

EPA: *Providing anaesthetic care for patients with severe pre-existing cardiac conditions*

- **I find the focus on "cardiac conditions" difficult, if so then airway, endocrinological etc. need to be addressed as well**
- Last part of training
- Valid for doctors outside of hospitals without integrated cardiothoracic department

We think that patients with preexisting cardiac conditions (and of course other patients, too) need special considerations, as anaesthesia might have great impact on the hemodynamic of a patient (especially the elderly patient). Other conditions, such as lung, endocrinological etc. are underlying competencies integrated in the other EPAs (ASA classification, lung separation). Therefore, these conditions are also adequately reflected in this curriculum. The adaptation of the provided care a.e. instrumentation, induction, selection of technique in patients with severe cardiac conditions is essential to provide safe and high quality of care and should therefore be reflected in an individual EPA. Looking at the results, this is supported by the high CVI of 0,96 and agreement of 96% that this EPA is essential for anaesthesiologyl training.

EPA: *Providing emergency care for critically injured and ill patients during in-house transfers and diagnostic procedures*

- After ICU-rotation
- Highest priority at a maximum care hospital
- **In house patient transfer is sufficient**
- Should be board certification standard

Due to the comments and our discussions, we merged this EPA with the EPA "*Performing in-house transfers of critically ill patients*" and extended the resulting EPA with "*and injured*". These two EPAs have many overlapping competencies, therefore the nesting of the competencies into a "bigger" EPA is justified. The merged EPA is called "*Performing in-house transfers of critically ill and injured patients*" and should be entrustd after 3,58 years of training.

EPA: *Providing perioperative coagulation management including interpretation and therapeutical consequences of thrombelastometry*

- This area of care is in constant flow and should not be limited to one singular lab system

EPA: *Management of in-house emergencies*

- Before board certification after intensive care rotation
- Depending on rotation plan

EPA: *Providing anaesthetic care for thoracic surgery (including lung seperation) with limited lung function*

- Last part of training

EPA: *Providing anaesthetic care and emergency management for critically injured and ill patients in the shock room*

- Last part of training
- More level 2

EPA: *Providing perioperative care for patients with major blood loss and pre-existing coagulation disorder*

- At a maximum care hospital yes, more attending task
- Without supervision leaning towards attending
- **Belongs to shock room and management of critically injured patients**

We do agree with the comment, that the competencies of this EPA do overlap with those of other EPAs and could be merged. However, we emphasize the relevance of coagulation management, especially when preexisting disorders are at hand. Therefore, we would not nest the competencies of this EPA into the other EPAs. This subject could be further discussed and adaptations and modifications could emerge.

EPA: *Providing anaesthetic care for pregnant patients with HELLP/pre-eclampsia/eclampsia*

- **Should be included in the care for pregnant patients**
- In the third year, latest in the fith year, depending on the structures of the hospital

We discussed merging this EPA with the EPA which describes the management of pregnant patients during labour- and decided that although the competencies do overlap- the significance of HELLP should be highlighted. Furthermore, 30% of HELLP occurs postpartum- the anaesthetist should have a certain level of awareness for this live threatening condition. Therefore we did not merge the EPAs.

EPA: *Providing perioperative care for critically injured patients with increased intracranial pressure*

- **The care for critically injured patients is mentioned often, I think EPAs should be merged and the number reduced. Intracranial pressure was mentioned at another place. It's especially relevant in critically injured, but does it need an extra EPA?**
- **Is already covered in critically injured and neurosurgery**

We think that these are valuable and significant comments- and we agree with them. Due to overlapping competencies in other EPAs we merged this EPA with the EPA "*Providing anaesthetic care for intracranial surgery with (the risk of) increased intracranial pressure*" and "*Providing anaesthetic care and emergency management for critically injured and ill patients in the shock room*".

EPA: *Administer general anaesthesia in pediatric patients under the age of five*

- **In don't think the age limit of five years makes sense. Under the age of 12 months/10 kg are special competencies (and a central care) reasonable, which cannot be taught to every trainee (because of the caseload). Offering a fellowship would be good. Any attending should be able to care for a child over the Age of 2.**
- More topic for attendings
- Last part of training
- Under two years after special rotation

We think that the age limit can be discussed. This distinction between under and over the age of five has been implemented in the German training for years. We do agree though that a further fragmentation of this EPA can be helpful in mastering the required competencies and reaching entrustment. The underlying competencies could be achieved, starting at a higher age (patients) and gradually moving to younger patients in the process. Further discussions would be interesting, involving all stakeholders.

EPA: *Providing anaesthetic and haemodynamic management of uterine atony*

- Simulation
- Topic for attendings
- Clinically dramatic emergency, needs to be mastered

EPA: *Providing perioperative care for patients undergoing cardiothoracic surgery*

- Specialized care, fellowship

- **Cardiothoracic surgery only belongs to my opinion in small parts into general training. Especially the time it takes to do cardioanaesthesia with TEE etc. independently is not there.**
- Independently as attending
- Last part of training
- **Really specialized and only at specialized centers necessary**
- **Cardiothoracic surgery should not be standard until the fifth year (also not provided everywhere)**
- **Depending on the hospital structure at least a theoretical curriculum for basics**
- **Depending on rotation and department**
- **Cardiothoracic surgery is an option, not a must! The third question cannot be answered, if I find this area as not necessary. In this case the possible answers year 1-5 are not relevant, which can't be expressed. A weakness in the questionnaire.**
- No year of training because not an EPA

This EPA was excluded from the final curriculum due to the low agreement of 64%, regarding the question if the EPA should be part of the curriculum. Furthermore, the CVI was also low with 0,48. We do agree with the comments that performing anaesthesia for cardiothoracic surgery is a special area that is not provided by every hospital and therefore should be part of a special fellowship during or after the basic training.

EPA: *Administer general anaesthesia in neonatal patients*

- **Specialized care, fellowship**

- Attending
- Topic for attendings
- **I would differentiate between over and under the age of 5**
- Depending on the hospital structure, more optional although relevant for basics of emergency care
- Depending on rotation plan and department
- **Compare to cardioshoracic surgery. In the fith year of training an independent care for neonatal patients can't be expected.**

This EPA was included in the final curriculum since it reached an acceptance of 80%. We do think that the low CVI of 0,68, is definitely a reason for further discussions. We believe that basic training of the competencies of this EPA should be provided in general training. More complex cases should be provided by adequately trained and equipped professionals at centers. Therefore, not all competencies of this EPA need to be conveyed and assessed for genera
